# BAMscale: quantification of next-generation sequencing peaks and generation of scaled coverage tracks

**DOI:** 10.1186/s13072-020-00343-x

**Published:** 2020-04-22

**Authors:** Lorinc S. Pongor, Jacob M. Gross, Roberto Vera Alvarez, Junko Murai, Sang-Min Jang, Hongliang Zhang, Christophe Redon, Haiqing Fu, Shar-Yin Huang, Bhushan Thakur, Adrian Baris, Leonardo Marino-Ramirez, David Landsman, Mirit I. Aladjem, Yves Pommier

**Affiliations:** 1 Developmental Therapeutics Branch and Laboratory of Molecular Pharmacology, Center for Cancer Research, National Cancer Institute, NIH, 37 Convent Dr, Bethesda, MD 20892 USA; 2 Computational Biology Branch, National Center for Biotechnology Information, National Library of Medicine, NIH, 8600 Rockville Pike, Bethesda, MD 20892 USA

**Keywords:** Histone modifications, Expression, ATAC-seq, ChIP-seq, NS-seq, Replication timing, Replication origins, RNA-seq, SLFN11

## Abstract

**Background:**

Next-generation sequencing allows genome-wide analysis of changes in chromatin states and gene expression. Data analysis of these increasingly used methods either requires multiple analysis steps, or extensive computational time. We sought to develop a tool for rapid quantification of sequencing peaks from diverse experimental sources and an efficient method to produce coverage tracks for accurate visualization that can be intuitively displayed and interpreted by experimentalists with minimal bioinformatics background. We demonstrate its strength and usability by integrating data from several types of sequencing approaches.

**Results:**

We have developed *BAMscale*, a one-step tool that processes a wide set of sequencing datasets. To demonstrate the usefulness of *BAMscale*, we analyzed multiple sequencing datasets from chromatin immunoprecipitation sequencing data (ChIP-seq), chromatin state change data (assay for transposase-accessible chromatin using sequencing: ATAC-seq, DNA double-strand break mapping sequencing: END-seq), DNA replication data (Okazaki fragments sequencing: OK-seq, nascent-strand sequencing: NS-seq, single-cell replication timing sequencing: scRepli-seq) and RNA-seq data. The outputs consist of raw and normalized peak scores (multiple normalizations) in text format and scaled bigWig coverage tracks that are directly accessible to data visualization programs. *BAMScale* also includes a visualization module facilitating direct, on-demand quantitative peak comparisons that can be used by experimentalists. Our tool can effectively analyze large sequencing datasets (~ 100 Gb size) in minutes, outperforming currently available tools.

**Conclusions:**

*BAMscale* accurately quantifies and normalizes identified peaks directly from BAM files, and creates coverage tracks for visualization in genome browsers. BAMScale can be implemented for a wide set of methods for calculating coverage tracks, including ChIP-seq and ATAC-seq, as well as methods that currently require specialized, separate tools for analyses, such as splice-aware RNA-seq, END-seq and OK-seq for which no dedicated software is available. BAMscale is freely available on github (https://github.com/ncbi/BAMscale).

## Background

Improved technologies and decreasing sequencing costs enable in-depth analyses of chromatin and gene expression changes for genome-wide comparisons. These integrative multi-omics studies elucidate the functionalities of coding and non-coding parts of the genome, their influence on development of complex disease such as cancers [1–4] and their translational implications [5–7].

Currently many studies focus on identifying protein–DNA interactions through sequencing (ChIP-seq) [8, 9]. By mapping protein-bound DNA, we can determine transcription factor binding sites or histone modification distributions across the genome. Other analyses focus on identifying open-chromatin and DNA-accessible regions [10–13], which are useful to classify enhancer regions, and transcription factor footprints [14–16]. Integrating these analyses with gene expression data such as RNA-seq [17–19], it is possible to gain better understanding of the architecture and regulation of the genome.

Recently, a new method has been introduced for genome-wide mapping of DNA double-strand breaks (END-seq) [20]. By enabling detection of DNA breaks that occur in a small fraction of a cell population, END-seq can be used to understand how breaks occur and are repaired.

To understand DNA replication patterns across the genome, next-generation sequencing methods are increasingly used. They are either based on sequencing newly synthesized and RNA-primed DNA, such as Okazaki fragment sequencing (OK-seq) [21] for the lagging strand or nascent-strand sequencing (NS-seq) for the leading strand [22]. These approaches are useful to pinpoint where DNA replication is initiated in the genome. The order of genome replication can also be measured with replication-timing sequencing, which involves identifying copy-number state differences between diploid G1-phase and replicating S-phase (or asynchronous—AS) cells [23–26].

Although sequencing methods are routinely used, data analyses need constant improvement to reduce the number of steps prone to error. In many cases, results are difficult to accurately reproduce because they are obtained with “in-house” scripts. One such example is the quantification of ChIP-seq/ATAC-seq peaks followed by normalization. Another example is generating sequencing coverage tracks [27–29], which requires either more computation time for scaling and/or multiple steps to get accurate results. Additionally, many sequencing types do not have dedicated solutions for creating coverage tracks for accurate visualization. One example is OK-seq, where replication fork directionality (RFD) is used to identify replication origins in the genome. RFD is calculated from the ratio of reads aligning on the forward and reverse strand, which is usually accomplished by calculations involving multiple steps. Another example is splice-aware RNA-seq, for which the coverage tracks can be calculated using multiple tools, but many of them disregard exon–intron boundaries are disregarded, yielding inaccurate representations of splicing events.

**Fig. 1:**
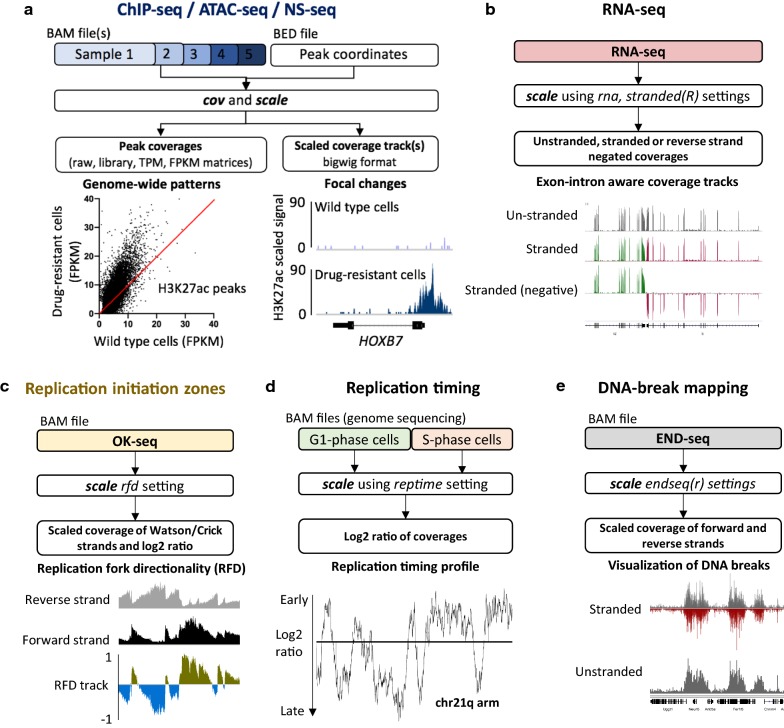
Application and benchmarking of *BAMscale* on different sequencing datasets. **a** Scaled coverage track generation and peak quantification of ChIP-seq and ATAC-seq data. Local differential H3K27ac signal at the HOXB7 locus in MV4-11 (wild type) and PKC412-resistant (R) (drug-resistant) cells, and global H3K27ac increase. **b** Creating exon–intron boundary aware stranded and un-stranded RNA-seq coverage tracks. **c** OK-seq coverage track creation using *BAMscale* outputs scaled strand-specific coverage tracks, and the replication-fork directionality tracks. **d** Analysis of replication timing data. **e** Mapping DNA-breaks from END-seq, creating strand-specific or unstranded coverage tracks

Here, we introduce *BAMscale* (summarized in Fig. 1 and Table 1), a new genomic software tool for generating normalized peak coverages and scaled sequencing coverage tracks in bigWig format. *BAMscale* is a one-step tool that processes DNA sequencing datasets to create scaled and normalized quantifications and coverage tracks. As summarized in Table 1, *BAMscale* can process sequencing data generated by diverse experimental approaches, including chromatin binding (ChIP-seq), chromatin accessibility (ATAC-seq), stranded and unstranded RNA-seq, DNA replication assays (OK-seq, NS-seq and replication timing) and DNA double-strand breaks sequencing (END-seq). We developed *BAMscale* in C-programming language using the *samtools* library [30] and *libBigWig* [27], achieving superior performance compared to existing tools. *BAMscale* can process 100 GB of aligned data (in BAM format) in under 20 min using a regular computer with 4 processing threads. To demonstrate the potential of *BAMscale*, we processed a wide set of sequencing datasets (Additional file 1: Table S1), benchmarking the performance with existing tools, paired with post-analyses. The tool, with installation and extensive usage examples, is available at https://github.com/ncbi/BAMscale.

**Table 1:** Capabilities of *BAMScale* and other publicly available tools

	Tool					
	*BAMscale*	*IGVtools*	*bedtools*	MACS (callpeak followed by bedgraph2bigwig (UCSC)**	MACS (pileup followed by bedgraph2bigwig (UCSC)**	*deeptools*
Creating coverage tracks						
ChIP-seq/ATAC-seq	X	X		X	*	X
ChIP-seq/ATAC-seq (normalized)	X			X		X
Log2 coverage (replication timing)	X					X
OK-seq (RFD calculation)	X					
RNA-seq	X	*		*	*	X
RNA-seq (splice-aware)	X					
Stranded coverage	X					X
Quantifying peaks						
Raw read counts	x		x			
Normalized read counts	x					

* Scaling factor cannot be specified

** BAM file has to be pre-filtered for alignment quality

## Results

Some of the most basic functions of *BAMscale* are the capability to quantify detected peaks and the ability to scale the sequencing coverage for visualization. *BAMScale* modules are available for processing data from BAM files generated by standard chromatin analyses such as ChIP-seq and ATAC-seq experiments and contains additional custom functions to process sequencing data from RNA-seq (rna, stranded or unstranded—Fig. 1b), OK-seq (rfd, Fig. 1c), replication timing analyses (reptime, Fig. 1d) and DNA break mapping (endseq(r), Fig. 1e). These modules allow direct quantification of peaks from various experimental sources, which are often tested for colocalization in chromatin analyses, using a single, uniform tool.

### Peak quantification and scaling coverage track from ATAC-seq data

**Fig. 2:**
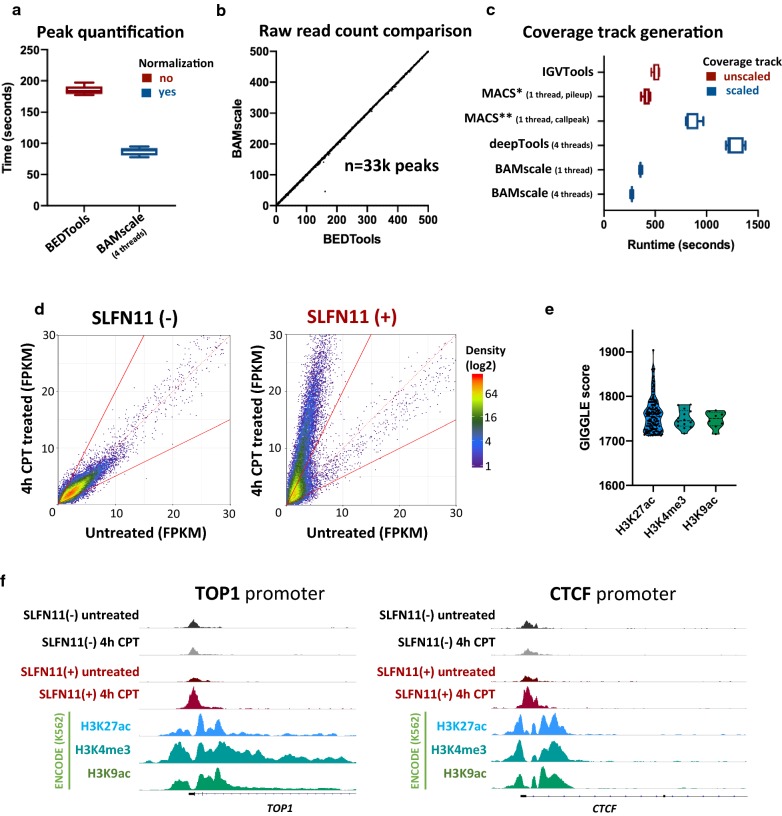
Benchmarking *BAMscale* on ATAC-seq data. **a** Performance comparison of peak quantification and **b** correlation of raw read counts in ~ 33 k peaks between *BAMscale* and bedtools. **c** Coverage tracks generation benchmarks using *IGVtools* (unscaled output), *MACS2* (pileup: unscaled, callpeak: scaled with peak calling), deeptools and *BAMscale* (scaled output). **d** ATAC-seq signal change induced from camptothecin (CPT) is observed in wild-type CEM-CCRF cells (SLFN11 positive), and not in the SLFN11 isogenic knockout. **e** Colocalization of opening ATAC-seq peaks using GIGGLE and Cistrome. **f** Examples of chromatin accessibility in the TOP1 and CTCF genes

To test the capabilities of *BAMScale*, we first implemented it to compare chromatin accessibility from ATAC-seq data in SLFN11-proficient and deficient cells [31]. While the performance of *BAMscale* for peak quantification was comparable to the most commonly used *BEDTools* [2] program with a single processing thread (Additional file 2: Fig. S1A), *BAMscale* reduced execution time by ~ 50% when using four threads (Fig. 2a). Minor reductions of execution time were observed using 8 threads (Additional file 2: Fig. S1A). Notably, *BEDTools* only calculates raw read counts, whereas *BAMscale* performs normalization of raw read counts while outputting FPKM, TPM and library size normalized peak scores. This enables a direct comparison of peaks between samples from cells with different genomic backgrounds undergoing diverse treatments. As shown in Fig. 2b and Additional file 2: Fig. S1B (each point represents one peak), correlations of raw read counts from the two methods were above 0.99 (Fig. 2b and Additional file 2: Fig.S1B), resulting in high density of points on the diagonal that give the appearance of a straight line. Out of the 32,819 quantified peaks, only a single ATAC-seq peak had low read counts from *BAMscale* and high read counts from *BEDTools*. That peak was covered predominantly by reads where the read-pair mapped to a different chromosome (Additional file 2: Fig. S1C) that were removed by default by *BAMscale*. The mean execution time to create sequencing coverage tracks with *BAMscale* was 4.6-fold faster than *deepTools bamCoverage* and 1.8-fold faster than *IGVtools* (which does not scale for library size). We have also attempted to compare execution times for this task with the *MACS2* program, which is able to create bedgraph formatted coverage tracks that need to be converted to bigwig format (Table 1). The pileup function of *MACS2* achieved similar run-times as *BAMscale*, but did not scale the coverage, whereas the callpeak function scaled coverages with slower run-times (3.1 × slower) due to concomitant peak calling (Fig. 2c).

We next compared the effect of the topoisomerase I (TOP1) inhibitor camptothecin (CPT) on ATAC-seq patterns in human leukemia CCRF-CEM (SLFN11-positive) cells and their isogenic SFLN11-knockout [31]. After CPT treatment, chromatin accessibility remained unchanged in the *SLFN11*-KO cells, while accessibility of pre-existing sites strongly increased in the *SLFN11*-positive cells (Fig. 2d). Using the *GIGGLE* tool [32] on the *Cistrome* [33] website, we found that ATAC-seq peaks strongly overlapped with H3K27ac, H3K4me3 and H3K9ac sites, which are histone marks associated with active genes (Fig. 2e). Colocalization analysis of sites with > threefold increase during CPT treatment in *SLFN11*-positive cells showed ~ 20% increase in overlap with H3K4me3 and H3K9ac sites, identified using *Coloweb* [34] (Additional file 1: Table S2, Additional file 2: Fig. S2). DNA accessibility sites were strongly enriched in gene promoter regions, such as in the *TOP1* and *CTCF* gene promoters (Fig. 2f).

*BAMscale* is designed to quantify ChIP-seq/ATAC-seq peaks from BAM and BED files, producing raw read counts, as well as TPM, FPKM and library size normalized peak scores (Fig. 1a). By providing accurate peak quantification in parallel with generated scaled coverage tracks, *BAMscale* simplifies the comparison and visualization of genome-wide and local changes. To illustrate this point, we reanalyzed published histone ChIP-seq data from MV4-11 cell line and their isogenic counterpart (MV4-11R) resistant to PKC412, a multi-target protein kinase inhibitor [35]. Using the *BAMscale* “cov” and “scale” functions, we accurately quantified peak strengths, and created scaled coverage tracks ready for visualization. In agreement with published results, we observed a global increase of H3K27ac, a decrease in H3K27me3 and a largely unchanged H3K4me3 signal in the drug-resistant cells (Fig. 1a, Additional file 2: Fig. S3A-C). Drug-resistant cells displayed elevated protein expression of HOXB7 [35], which has increased histone H3K27ac signal, a known marker for active genes.

### RNA-seq data coverage track generation

**Fig. 3:**
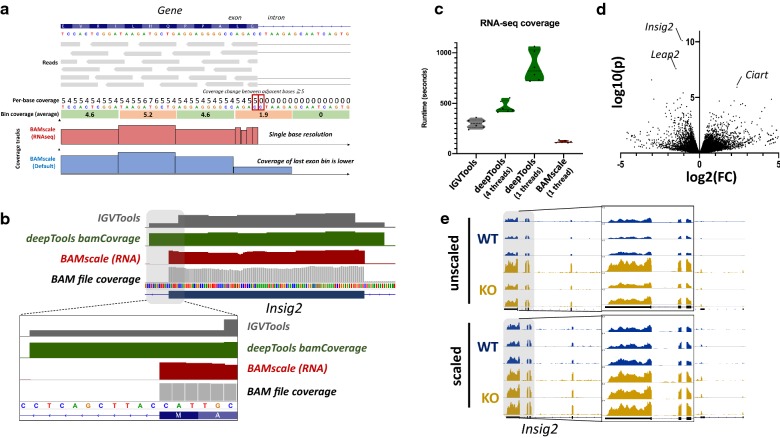
Re-analysis of RNA-seq data. **a** Overview of *BAMscale* RNA-seq mode. Coverage resolution is switched to single-base resolution in bins where adjacent base coverages exceed 4 reads. **b** Comparison of coverage tracks between *IGVTools*, *Deeptools* and *BAMscale*. **c** Performance comparison of three tools. **d** Differentially expressed genes between Top1mt wild-type and knock-out mice. **e** Comparison of unscaled and scaled coverages

RNA-seq involves sequencing of mature RNA, where introns are spliced-out of the molecules. For this reason, genome alignment of RNA-seq is performed with splice-aware aligners such as STAR [36] or HISAT2 [37]. These tools are able to split sequencing reads between two (or more) exons with or without prior gene annotations. Currently most tools that generate coverage tracks (in bigWig or tdf format) are capable of identifying splicing events in the alignments, but their binning process creates inaccurate representations. This causes strong coverage drops in bins that overlap exon–intron boundaries. For this reason, we implemented an RNA-seq-compatible function for *BAMscale***(**Fig. 1b). Compared to the standard run, in RNA-seq mode *BAMscale* searches for sudden changes in coverages between adjacent bases in a bin (usually >=5 reads), where resolution changes from bin to a single base pair (illustrated in Fig. 3a). A major advantage of this method is that no gene/transcript annotation is needed for accurate representation of recurrent splicing events. Output coverages can be set to be unstranded (“rna” operation) or stranded (operation set to “stranded” or “stranded”), where two separate bigWig files are created for the two strands (Additional file 2: Fig. S4).

To test the potential of *BAMscale* RNA-seq mode, we reprocessed previously published RNA-seq data from *Top1mt* wild type and knock-out mice [38]. *BAMscale* is capable of producing more accurate, single-base resolution tracks at exon–intron boundaries, compared to *IGVTools* or *deepTools bamCoverage* (Fig. 3b). Additionally, the RNA-seq compatible *BAMscale* (using one processing thread) is 2.5-fold faster than *IGVTools*, 7.2-fold and 3.9-fold faster than *deepTools bamCoverage* running on one or four threads, respectively (Fig. 3c). After differential expression analysis, we identified several genes that are upregulated in the KO samples (Additional file 1: Table S3), such as *Insig2*, which was the statistically most significantly, but with a moderate fold-change increase around 2x (Fig. 3d). This subtle change in expression is somewhat visible in the unscaled tracks, but the variation in the signal is very strong across replicates (Fig. 3e, upper tracks). This can be overcome by either extracting scaling factors for samples from the differential expression analysis program such as *DESeq* *2* (Fig. 3e, lower tracks), or by using the genome-size scaling, which scales to the number of sequenced bases. These methods ensure more comparable results for visualization by reducing the variations due to sequencing library size differences.

### Alignment of DNA-breakage sites and replication origins with replication timing domains

*BAMScale* expands the range of data type that can be quantitatively scaled and analyzed to include OK-seq, replication timing and splice-aware RNA-seq analyses in addition to ChIP-seq/ATAC-seq, which can be analyzed by other tools as well (see Table 1 for a detailed comparison of data types and analysis tools). To test these capabilities, we processed replication timing, OK-seq and END-seq data derived from activated mouse B cells [39], where the coverage tracks for the three datasets were created with *BAMscale.*

Replication timing sequencing calculates the order of genome replication. This usually involves the comparison of sequencing depths between G1-phase and S-phase cells. Replication timing log2 coverages of two BAM files can be calculated with *BAMscale* by setting the “reptime” flag as the operation. In this process, *BAMscale* first calculates the bin-level coverage of the genome for both BAM files, followed by separate signal smoothening. By default, the bin size is set to 100 bp, while the smoothening is set to 500 bins. After smoothening the coverage of the two input files, the log2 coverage is calculated and exported to a bigWig file for visualization.

For OK-seq, the replication fork directionality (RFD) can be calculated with *BAMscale*, for which no other dedicated tools are currently available. When *BAMscale* is set with the “rfd” operation, it calculates the bin-level coverage of the genome for reads aligning to the forward strand, and reverse strand separately, followed by RFD calculation of each bin [21]. In case of mapping of DNA breaks with END-seq, stranded bin-level coverages can be calculated by setting the operation flag to “endseq” (both strands have positive values) or “endseqr” (negative strand coverage will be negative) which allows to overlay the two strand coverages in one figure.

**Fig. 4:**
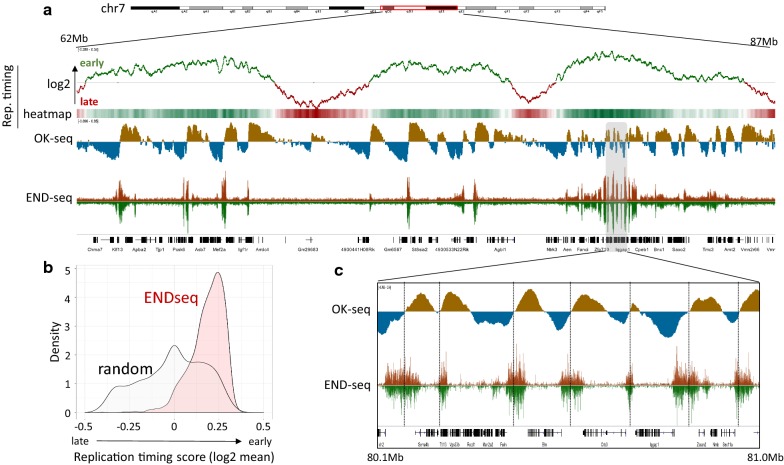
Comparison of replication timing and DNA-breaks. **a** Visualization of DNA-replication timing, synchronized replication origins (OK-seq) and DNA-breaks mapped with END-seq on chromosome 7. **b** Mean replication timing distributions of genomic regions enriched with END-seq signal compared to random peaks. **c** Elevated END-seq regions overlap early replicating regions

Visual comparison showed high similarly with the deposited tracks (Additional file 2: Fig. S5). As previously reported, END-seq DNA-break signals were predominantly observed in early replicating regions of the genome (Fig. 4a). Genome regions with stronger END-seq signal displayed a higher replication timing average calculated from the log_2_ tracks compared to randomly selected regions (Fig. 4b). Comparison of negative to positive strand switching in the replication initiation zones identified by OK-seq showed strong overlaps among regions with increased END-seq signal (Fig. 4c).

**Fig. 5:**
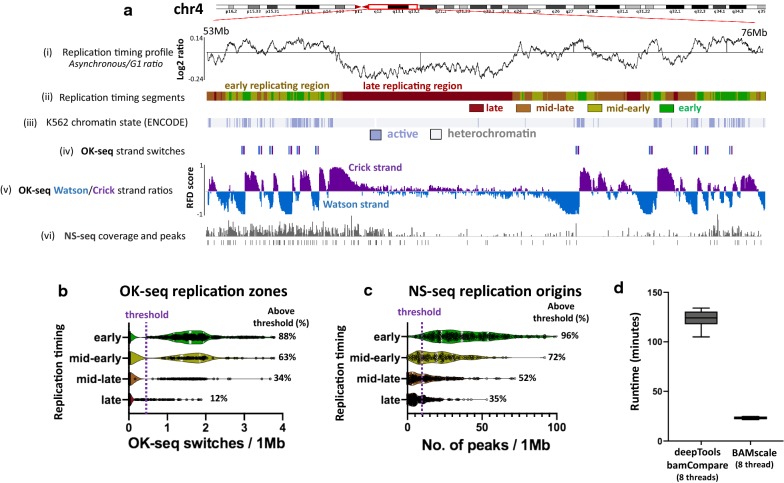
Comparison of different replication sequencing methods. **a** Replication timing ratios where higher ratios correspond to earlier replicating regions (i), replication timing profile (ii), active/repressed chromatin regions (iii), strong OK-seq strand switch coordinates (iv), OK-seq replication-fork directionality ratios (v) and NS-seq (replication origin) tracks (vi) for K562 cell line. **b** OK-seq strand switches in the four segments of replication. **c** NS-seq peak abundances in the four replication timing phases. **d** Comparison of analysis time between *deeptools bamCompare* and *BAMscale* for replication timing data

Finally, we compared replication timing data to OK-seq and NS-seq (Nascent strand sequencing) data from the human leukemia K562 cell line. Replication timing results (Fig. 5a(i)) and the generated segments (Fig. 5a(ii)) showed that early-replicating regions strongly correlate with active chromatin regions (Fig. 5a(iii)) identified with *ChromHMM* [40, 41]. Furthermore, *BAMscale* also showed a strong overlap of OK-seq [42]. RFD strand switches (associated with synchronized replication initiation zones) with active euchromatin (Fig. 5a(iv, v)). Fewer than 0.5% of identified OK-seq strand switches were identified in heterochromatin, where no overlap with active chromatin regions was found. Similarly, we observed higher NS-seq signal (and replication origin peaks) in euchromatin (Fig. 5a(vi). Early-replicating regions tend to be associated with more replication initiation sites, which gradually decrease in later phases of replication timing (Fig. 5b). These results correlate strongly with the NS-seq results showing that early replicating regions have higher peak densities compared to late-replicating regions [43] (Fig. 5b). We also tested *BAMscale* on 80 single-cell replication timing sequencing (scRepli-seq) samples [44]. We were able to accurately reproduce the single-cell log2 replication timing profiles from G1 phase and mid-S phase cells (Additional file 2: Fig. S6), requiring on average 11 s of processing time for each sample pair using 4 processing threads. Furthermore, we compared the performance of *BAMscale* and *deepTools bamCompare* on the replication timing data derived from the human leukemia K562 cell line using eight processing threads. The sequencing data consists of > 103 Gb of sequencing data in BAM format, which we re-analyzed six times. The mean run time of *BAMscale* was 23.2 min, which is a 5.3-fold decrease in analysis time, compared to 123.1 min required for *deepTools*.

## Discussion

Widespread usage of DNA and RNA capture-based methods helps us understand and categorize changes in chromatin state and their regulatory effects on DNA replication and gene expression. Visualization of genome-wide data is a crucial step to identify complex genomic patterns and relationships. Because of the increased usage of next-generation sequencing both in basic research and clinical settings, it is important to analyze data reproducibly by removing as many analysis steps as possible, as they may be prone to error and be limiting for experimentalists.

*BAMscale* addresses two critical prevalent issues that are often encountered in sequencing-based chromatin analyses. First, since the scope of next-generation sequencing is usually genome-wide, the signal distribution of these techniques is generally visualized with different genomic viewers [29, 45]. However, available tools for sequencing track generation either require multiple steps or need long computation time to produce results ready for visualization [27–29]. Additionally, quantification and normalization of ChIP-seq and ATAC-seq peak strengths require multiple analysis steps using time-consuming, case-by-case programming of “in-house” scripts, i.e., time-consuming case-by-case programming. Second, although there are multiple tools to analyze genome-level coverage of sequencing data (e.g., *IGVTools* [29], *deepTools* [27], and *MACS2* [46] coverage mode coverage mode and *align2rawsignal* [https://code.google.com/p/align2rawsignal/]; Table 1), many sequencing approaches require specific analysis methods for accurate representations. A simple example is RNA-seq, where the binning process has to be splice-aware for accurate representation of exon–intron boundaries. These are currently not supported by the above-mentioned tools. Another example is OK-seq, which can be used to identify replication origins based on the calculation of replication fork directionality (RFD), for which no dedicated software is available. *BAMscale* provides a uniform, single-step scaling function for these diverse data types, including easy-to-use custom scripts that facilitate quantification and analyses. The additional post-analysis and visualization scripts of BAMscale allow experimentalists to compare, quantify and analyze data from a variety of experimental approaches, aiding in the integration of epigenetic studies.

We developed *BAMscale* to analyze data in a quick, simple and reproducible manner. It is developed in basic C-programming, resulting in very fast execution times compared to previous methods. To facilitate data analysis, we implemented multiple pre-defined settings for a wide set of sequencing types accompanied with extensive tutorials (https://github.com/ncbi/BAMscale wiki page).

Using *BAMscale* as a peak quantification method and a scaled coverage-track generation tool, users can identify single focal changes in the genome as well as understand how certain conditions alter global chromatin. We have also implemented an RNA-seq-compatible version enabling accurate visualization of exon–intron boundaries from both stranded and un-stranded data. Notably, *BAMscale* eliminates the need to perform and convert outputs from multiple analyses tools to quantify and visualize data measuring chromatin modifications (ChIP-seq/ATAC-seq), transcription (splice-aware RNA-seq), DNA breakage (END-Seq) and replication (OK-seq, replication timing, NS-Seq). This capability facilitates the analyses of the effects of perturbation on those concomitant chromatin transactions and provides a methodological basis to address important issues such as the coordination of DNA replication and transcription or the orchestration of DNA damage repair with histone modifications. Integration of these multiple analyses would also enable the stratification and identification of genomic regions of interest displaying alterations in one or multiple epigenetic properties.

## Conclusions

*BAMscale* is a tool that can be used to accurately quantify and normalize identified peaks directly from BAM files, as well as create coverage tracks for visualization in genome browsers. The uniform scaling function and the peak-size comparison visualization tool allow easy interpretation of data from various sequencing approaches by experimentalists, and the availability of custom scripts facilitate the integration of distinct chromatin interactions interrogated with diverse methodologies. Due to the multithreaded implementation, our tool outperforms currently used methods. We implemented sequencing-specific coverage track calculation modes including: (1) replication timing, (2) replication fork directionality analysis from OK-seq data, (3) strand-specific coverage of DNA breaks from END-seq and (4) splice-aware RNA-seq coverage modes, many of which lack any dedicated software. *BAMscale* is freely available on github (https://github.com/ncbi/BAMscale).

## Methods

### BAMscale algorithm

#### Peak quantification

Peaks can be quantified with *BAMscale*’s *cov* function, which takes as input a BED file with peak coordinates, and one or multiple BAM files, outputting raw read counts, FPKM, TPM and library size normalized peak scores. Paired-end reads can be quantified in two main ways: (1) using each read as a single entity, or (2) counting read pairs as one fragment. Additionally, it is possible to count reads that follow either the strand direction of each peak in the BED file, or simply calculate forward or reverse reads only.

During peak quantification, *BAMscale* by default first reads the entire BAM file(s) to count the number of aligned reads using the selected alignment filters to get the effective library size. This approach gives more accurate alignment statistics than using the BAM index file, which has information on number of aligned reads only, containing duplicate reads and low-quality reads as well. After calculating the effective library size, *BAMscale* counts the number of overlapping reads with each coordinate in the BAM file, followed by FPKM [47], TPM [48] and library size (scaled to the smallest library) normalization.

To facilitate pairwise comparisons (as seen in Fig. 2d), we prepared an interactive R script (available at https://github.com/ncbi/BAMscale) using the shiny, ggplot2, tidyr, ggrepel and gridExtra libraries to plot density dot-plots of the quantified (TPM, FPKM and library size normalized) peaks outputted from *BAMscale*.

#### Creating coverage tracks from sequencing data

To generate normalized coverage tracks, the *BAMscale* “scale” function first imports the coverage of every bin (changeable) of the genome, followed by either genome size scaling (based on the length of the genome), or read count scaling. During genome size scaling, the scaling factor is calculated by dividing the total number of aligned bases with the genome size, which is obtained from the header of the BAM file. In cases where the number of bases exceeds the genome size, scaling will reduce the per-bin coverage, while increasing the coverage when the sequenced bases are less than the genome size. The advantage of this approach is that each sample can be scaled separately. Alternatively, it is possible to scale multiple samples based on the library size. In these cases, the number of aligned reads is calculated for each sample, and scaling is done by scaling each sample to the smallest in the set. A drawback of this approach is that all samples have to be processed in parallel, which increases memory requirements (~ 500 Mb for each sample when the bin size is set to 5 bp). Additionally, it is possible to supply a scaling factor for each sample that will be used to adjust the coverages.

We implemented an RNA-seq-compatible option for creating coverage tracks with a difference in binning strategy. In RNA-seq mode, at cases where two adjacent bases in one bin have a coverage difference above 4 reads, the resolution is automatically changed to single base resolution. This enables the accurate representation of exon–intron boundaries.

Additionally, we implemented a signal smoothing option for coverage tracks. When smoothening a signal, the number of adjacent bins can be specified, which will be used to calculate the final signal of each bin.

In cases where two files are specified, different operations can be performed, such as calculating the log2 ratio of bins, or subtracting the values of bins.

For ease of use, we implemented predetermined settings to analyze replication timing data and END-seq data. In case of the “reptime” operation, the log2 coverage of two bam files are calculated for 100 bp bin sizes, with signal smoothening set to 500 bins. In case of END-seq, we can set the operation to “endseq”, which creates stranded coverage tracks, or “endseqr”, which negates the coverage track of the negative strand for ease of visualization.

#### OK-seq data and replication fork directionality

In case of OK-seq data first the Watson (forward) and Crick (reverse) strand coverages are calculated consecutively. After importing the strand-specific coverages, the replication fork directionality (RFD) is calculated as:

$$RFD_{i} = \frac{{X_{{{\text{Crick}}, i}} - X_{{{\text{Watson}},i}} }}{{X_{{{\text{Crick}},i}} + X_{{{\text{Watson}},i}} }},$$

where $X_{{{\text{Crick}}, i}}$ denotes the Crick read counts, $X_{{{\text{Watson}},i}}$ denotes the Watson read counts for the i-th bin (based on [21]).

### Sequenced data

#### NS-seq and replication timing sequencing of K562 cell lines

To produce high coverage sequencing of replication origins for the K562 human bone marrow derived cell line, we performed nascent-strand sequencing and replication timing sequencing available at GEO (GSE131417).

#### K562 NS-seq sample preparation

Replication origins were mapped using the nascent-strand sequencing and abundance assay [22]. Briefly, DNA fractionation was performed using a 5-30% sucrose gradient to collect DNA fractions ranging from 0.5 to 2 kb. Five prime single-strand DNA ends were phosphorylated by T4 polynucleotide kinase (T4PK) (NEB, M0201S). After phenol/chloroform treatment to remove T4 PK, DNA was precipitated, resuspended and then treated with lambda-exonuclease (NEB, M0262S) to remove genomic DNA fragments that lacked the phosphorylated RNA primer. After RNase treatment and DNA purification (Qiagen PCR purification kit, 28004), single-stranded nascent strands were random-primed using the Klenow and DNA Prime Labeling System (Invitrogen, 18187013). Double-stranded nascent DNA (1 μg) was sequenced using the Genome Analyzer II (Illumina).

#### K562 replication timing sample preparation

To get the pure G1 phase cells, 1 × 108 K562 cells were washed twice with cold PBS and fractionated by elutriation at each of the following flow speeds: 15 × 2, 16 × 2, 17 × 2, 18 × 2 19 × 2, 20 × 2 ml/min. Each fraction was stained by DAPI in PBS and confirmed by FACS. Genomic DNA from G1 phase and asynchronized K562 cells was extracted simultaneously according to manufacturer’s instructions (Qiagen, 69506).

### Data analysis

We demonstrate the capabilities of *BAMscale* on a wide set of sequencing datasets, such as ATAC-seq [31], ChIP-seq [35], replication timing data and END-seq [39], OK-seq [39, 42], single-cell Repli-seq and BrdU-IP [44] and stranded RNA-seq [38]. The complete list of analyzed samples, genome version and tissues of origins used in this study are shown in Additional file 1: Table S1.

#### Alignment of sequencing data

The next-generation sequencing data GEO/ENA and SRA ids, along with sample type, aligner (and version) and genome build summarizing 27 processed sequencing experiments can be found in Additional file 1: Table S1. ATAC-seq, ChIP-seq, OK-seq, END-seq and NS-seq were aligned with the *bwa mem* aligner [49] (version 0.7.17) or *dragen* pipeline [50] in case of the K562 replication timing. RNA-seq data were aligned using the *STAR* aligner [36] two-pass mode. Alignment settings were based on “best recall at base and read level” as shown in supplementary Table 37 of [51] to obtain the best alignments. Aligned (unsorted) reads were sorted using *samtools* [30] (version 1.8), followed by duplicate marking using picard-tools (version 2.9.2).

#### Peak calling and coverage track generation for ATAC-seq, ChIP-seq and NS-seq

Peaks were identified using MACS [46] peak caller (version 2.1.1.20160309), using the nomodel setting for ATAC-seq and NS-seq, and FDR set to 0.01 to filter low-quality peaks. ATAC-seq peaks were called using the narrow setting, while histone peaks were called using the broad peak setting of *MACS*. In case of NS-seq data, both broad and narrow peaks were called, the top 10% of narrow peaks were intersected with the broad peaks to retrieve the highest scoring regions. Called peaks for each cell line and condition were sorted and merged using *BEDTools* [28] (version 2.27.1). Peak quantification was performed with the “cov” function of *BAMscale* separately for each sequencing type.

#### Gene expression quantification and differential expression analysis from RNA-seq data

Raw read counts for each gene were calculated using the *TPMcalculator* program [52]. Differential expression analysis between wild-type and KO samples were calculated using *DESeq* *2* [53], where, as suggested in the manual, genes with less than ten reads on average were removed from the analysis. Scaling factors for each sample were obtained using *DESeq* *2 sizeFactors()* function. Scaled coverages were created with *BAMscale* “scale” function with operation set to “strandrnaR” and bin size set to 15 bases, scaling set to “-k custom” and scaling factor set to the reciprocal estimated factors from DESeq 2 for each sample.

#### OK-seq data

BigWig signal of aligned OK-seq reads were created with the *BAMscale* “scale” function, with the operation set to “rfd” (replication fork directionality), all other parameters were set to default.

#### END-seq data

The strand-specific coverage tracks for END-seq data were created the *BAMscale* “scale” function, with the operation set to “endseqr”. Two coverage tracks are created, one with the forward strand, and a separate track for negative strand reads, where the score is negated.

#### Replication timing data

Replication timing log2 ratio coverage tracks were created with the *BAMscale* “scale” function, with the operation set to “reptime”. The first specified BAM file is the G1-phase-specific sequencing data, the second BAM file was the asynchronous cell-cycle BAM file. Replication-timing segments were identified the “Replication_timing_segmenter.R” script developed in R, and deposited on github along the BAMscale code.

#### Single-cell (sc) Repli-seq

Replication timing log2 ratio coverage tracks for single-cell replication timing data [44] were created with the *BAMscale* “scale” function. The operation parameter was set to log2, and to reproduce the original analysis results, we set the bin size was set to 50 kb, and signal smoothening to 4 (resulting in 400 kb smoothening). In case of the standard CBA/MsM samples the “CBMS1_ESC_single_G1_01” (GSM2904978) sample was used as the G1 phase reference, and sample “CBMS1_Day7Diff_ESC_single_G1_01” (GSM2905031) was used as the G1 phase reference for the 7-day differentiated CBA/MsM samples.

#### BrdU-IP replication timing data

The log2 coverage of early and late S-phase BrdU-IP sequencing was calculated using the *BAMscale* “scale” function. Similarly to the scRepli-seq data, the operation parameter was set to log2, and to reproduce the original analysis results, we set the bin size to 50 kb, and signal smoothening to 4 (resulting in 400 kb smoothening).

## Supplementary information

**Additional file 1: Table S1.** Detailed list of processed samples. **Table S2.** Colocalization statistics of ATAC-seq peaks with > 3x opening and < 3x opening induced by camptothecin (CPT) treatment in human leukemia CCRF-CEM SLFN11 wild type and KO. **Table S3.** Differential expression analysis results between Top1mt wild type and knockout murine liver tumor samples.

**Additional file 2. Fig.** **S1.** Benchmarking and comparison of different tools using ATAC-seq data. A) Peak quantification performance using *bedtools* and *BAMscale* (1, 4 and 8 execution threads). B) Comparison of raw read counts between *bedtools* and *BAMscale* in six ATAC-seq samples. C) IGV screenshot of a peak overestimated by *bedtools* in all samples, where read pairs align to different chromosomes. **Fig. S2.** Colocalization of ATAC-seq peaks. Peaks with > threefold opening had increased colocalization with H3K4me3 and H3K9ac compared to peaks with weaker or no increase. **Fig. S3.** Changes in histone ChIP-seq signal between MV4-11 and MV4-11R cells. A) H3K27me3 signal decreased, B) H3K27ac signal increased, and C) H3K4me3 signal did not change in the MV4-11R cells compared to the MV4-11 cells. **Fig.** **S4.** Stranded and unstranded RNA-seq coverage tracks created with *BAMscale*. **Fig. S5.** Comparison of deposited END-seq and OK-seq data reprocessed with *BAMscale*.

## Data Availability

BAMscale is freely available on github (https://github.com/ncbi/BAMscale). All re-analyzed samples (including the GEO and SRA identifiers) are summarized in Additional file 1: Table S1. The K562 cell line NS-seq and replication timing data are available at GEO (GSE131417).

## Abbreviations

ATAC-seq: Assay for transposase-accessible chromatin using sequencing
BAM: Binary simple alignment format
BED: Browser extensible data
ChIP-seq: Chromatin immunoprecipitation followed by sequencing
CPT: Camptothecin
END-seq: DNA double-strand break mapping sequencing
FPKM: Fragments per kilobase of transcript per million mapped reads
NS-seq: Nascent-strand sequencing
OK-seq: Okazaki fragments sequencing
RFD: Replication fork directionality
TPM: Transcripts per million

## References

[1] Huang YH (2018). POU2F3 is a master regulator of a tuft cell-like variant of small cell lung cancer. Genes Dev.

[2] Borromeo MD (2016). ASCL1 and NEUROD1 reveal heterogeneity in pulmonary neuroendocrine tumors and regulate distinct genetic programs. Cell Rep.

[3] Bernt KM (2011). MLL-rearranged leukemia is dependent on aberrant H3K79 methylation by DOT1L. Cancer Cell.

[4] Jang SM (2018). The replication initiation determinant protein (RepID) modulates replication by recruiting CUL4 to chromatin. Nat Commun.

[5] Patten DK (2018). Enhancer mapping uncovers phenotypic heterogeneity and evolution in patients with luminal breast cancer. Nat Med.

[6] Raisner R (2018). Enhancer activity requires CBP/P300 bromodomain-dependent histone H3K27 acetylation. Cell Rep.

[7] Ross-Innes CS (2012). Differential oestrogen receptor binding is associated with clinical outcome in breast cancer. Nature.

[8] Johnson DS (2007). Genome-wide mapping of in vivo protein-DNA interactions. Science.

[9] Barski A (2007). High-resolution profiling of histone methylations in the human genome. Cell.

[10] Buenrostro JD (2015). ATAC-seq: a method for assaying chromatin accessibility genome-wide. Curr Protoc Mol Biol.

[11] Song L, Crawford GE (2010). DNase-seq: a high-resolution technique for mapping active gene regulatory elements across the genome from mammalian cells. Cold Spring Harb Protoc.

[12] Giresi PG (2007). FAIRE (Formaldehyde-Assisted Isolation of Regulatory Elements) isolates active regulatory elements from human chromatin. Genome Res.

[13] Boyle AP (2008). High-resolution mapping and characterization of open chromatin across the genome. Cell.

[14] Davie K (2015). Discovery of transcription factors and regulatory regions driving in vivo tumor development by ATAC-seq and FAIRE-seq open chromatin profiling. PLoS Genet.

[15] Lu Z (2017). Combining ATAC-seq with nuclei sorting for discovery of cis-regulatory regions in plant genomes. Nucleic Acids Res.

[16] Baek S, Goldstein I, Hager GL (2017). Bivariate genomic footprinting detects changes in transcription factor activity. Cell Rep.

[17] Lister R (2008). Highly integrated single-base resolution maps of the epigenome in Arabidopsis. Cell.

[18] Mortazavi A (2008). Mapping and quantifying mammalian transcriptomes by RNA-Seq. Nat Methods.

[19] Nagalakshmi U (2008). The transcriptional landscape of the yeast genome defined by RNA sequencing. Science.

[20] Canela A (2016). DNA breaks and end resection measured genome-wide by end sequencing. Mol Cell.

[21] Petryk N (2016). Replication landscape of the human genome. Nat Commun.

[22] Martin MM (2011). Genome-wide depletion of replication initiation events in highly transcribed regions. Genome Res.

[23] Marchal C (2018). Genome-wide analysis of replication timing by next-generation sequencing with E/L Repli-seq. Nat Protoc.

[24] Mukhopadhyay R (2014). Allele-specific genome-wide profiling in human primary erythroblasts reveal replication program organization. PLoS Genet.

[25] Hansen RS (2010). Sequencing newly replicated DNA reveals widespread plasticity in human replication timing. Proc Natl Acad Sci USA.

[26] Koren A (2014). Genetic variation in human DNA replication timing. Cell.

[27] Ramirez F (2014). deepTools: a flexible platform for exploring deep-sequencing data. Nucleic Acids Res.

[28] Quinlan AR, Hall IM (2010). BEDTools: a flexible suite of utilities for comparing genomic features. Bioinformatics.

[29] Robinson JT (2011). Integrative genomics viewer. Nat Biotechnol.

[30] Li H (2009). The sequence alignment/map format and SAMtools. Bioinformatics.

[31] Murai J (2018). SLFN11 blocks stressed replication forks independently of ATR. Mol Cell.

[32] Layer RM (2018). GIGGLE: a search engine for large-scale integrated genome analysis. Nat Methods.

[33] Liu T (2011). Cistrome: an integrative platform for transcriptional regulation studies. Genome Biol.

[34] Kim R (2015). ColoWeb: a resource for analysis of colocalization of genomic features. BMC Genomics.

[35] Gollner S (2017). Loss of the histone methyltransferase EZH2 induces resistance to multiple drugs in acute myeloid leukemia. Nat Med.

[36] Dobin A (2013). STAR: ultrafast universal RNA-seq aligner. Bioinformatics.

[37] Kim D, Langmead B, Salzberg SL (2015). HISAT: a fast spliced aligner with low memory requirements. Nat Methods.

[38] Baechler SA (2019). The mitochondrial type IB topoisomerase drives mitochondrial translation and carcinogenesis. Nat Commun.

[39] Tubbs A (2018). Dual roles of poly(dA:dT) tracts in replication initiation and fork collapse. Cell.

[40] Ernst J, Kellis M (2012). ChromHMM: automating chromatin-state discovery and characterization. Nat Methods.

[41] Hoffman MM (2013). Integrative annotation of chromatin elements from ENCODE data. Nucleic Acids Res.

[42] Wu X (2018). Developmental and cancer-associated plasticity of DNA replication preferentially targets GC-poor, lowly expressed and late-replicating regions. Nucleic Acids Res.

[43] Smith OK (2016). Distinct epigenetic features of differentiation-regulated replication origins. Epigenet Chromatin.

[44] Takahashi S (2019). Genome-wide stability of the DNA replication program in single mammalian cells. Nat Genet.

[45] Haeussler M (2019). The UCSC genome browser database: 2019 update. Nucleic Acids Res.

[46] Zhang Y (2008). Model-based analysis of ChIP-Seq (MACS). Genome Biol.

[47] Trapnell C (2010). Transcript assembly and quantification by RNA-Seq reveals unannotated transcripts and isoform switching during cell differentiation. Nat Biotechnol.

[48] Wagner GP, Kin K, Lynch VJ (2012). Measurement of mRNA abundance using RNA-seq data: RPKM measure is inconsistent among samples. Theory Biosci.

[49] Li H, Durbin R (2009). Fast and accurate short read alignment with Burrows-Wheeler transform. Bioinformatics.

[50] Miller NA (2015). A 26-hour system of highly sensitive whole genome sequencing for emergency management of genetic diseases. Genome Med.

[51] Baruzzo G (2017). Simulation-based comprehensive benchmarking of RNA-seq aligners. Nat Methods.

[52] Vera Alvarez R (2019). TPMCalculator: one-step software to quantify mRNA abundance of genomic features. Bioinformatics.

[53] Love MI, Huber W, Anders S (2014). Moderated estimation of fold change and dispersion for RNA-seq data with DESeq2. Genome Biol.

