# Supplementary material for: BAMscale: quantification of next-generation sequencing peaks and generation of scaled coverage tracks
Source: Epigenetics Chromatin. 2020 Apr 22;13:21. doi: 10.1186/s13072-020-00343-x (PMC7175505; doi:10.1186/s13072-020-00343-x)
Supplement: Supplementary file 2 — Additional file 2. Fig. S1. Benchmarking and comparison of different tools using ATAC-seq data. A) Peak quantification performance using bedtools and BAMscale (1, 4 and 8 execution threads). B) Comparison of raw read counts between bedtools and BAMscale in six ATAC-seq samples. C) IGV screenshot of a peak overestimated by bedtools in all samples, where read pairs align to different chromosomes. Fig. S2. Colocalization of ATAC-seq peaks. Peaks with > threefold opening had increased colocalization with H3K4me3 and H3K9ac compared to peaks with weaker or no increase. Fig. S3. Changes in histone ChIP-seq signal between MV4-11 and MV4-11R cells. A) H3K27me3 signal decreased, B) H3K27ac signal increased, and C) H3K4me3 signal did not change in the MV4-11R cells compared to the MV4-11 cells. Fig. S4. Stranded and unstranded RNA-seq coverage tracks created with BAMscale. Fig. S5. Comparison of deposited END-seq and OK-seq data reprocessed with BAMscale. [file 13072_2020_343_MOESM2_ESM.pdf]

BAMscale: quantification of next-generation sequencing peaks and generation of scaled coverage tracks

Lorinc S. Pongor[1#], Jacob M. Gross[1], Roberto Vera Alvarez[2], Junko Murai[1], Sang-Min Jang[1], Hongliang Zhang[1], Christophe Redon[1], Haiqing Fu[1], Shar-Yin Huang[1], Bhushan Thakur[1], Adrian Baris[1], Leonardo Marino-Ramirez[2], David Landsman[2], Mirit I. Aladjem[1#], Yves Pommier[1#]

1. Developmental Therapeutics Branch and Laboratory of Molecular Pharmacology, Center for Cancer Research, National Cancer Institute, NIH, Bethesda, MD, USA
2. Computational Biology Branch, National Center for Biotechnology Information, National Library of Medicine, National Institutes of Health, Bethesda, MD, USA

# Corresponding authors: lorinc.pongor@nih.gov; aladjemm@mail.nih.gov; pommier@nih.gov

SUPPLEMENTARY TABLES LEGENDS

Supplementary table 1. Detailed list of processed samples.

Supplementary table 2. Colocalization statistics of ATAC-seq peaks with >3x opening and <3x opening induced by camptothecin (CPT) treatment in human leukemia CCRF-CEM SLFN11 wild type and KO.

Supplementary table 3. Differential expression analysis results between *Top1mt* wild type and knockout murine liver tumor samples.

SUPPLEMENTARY FIGURES

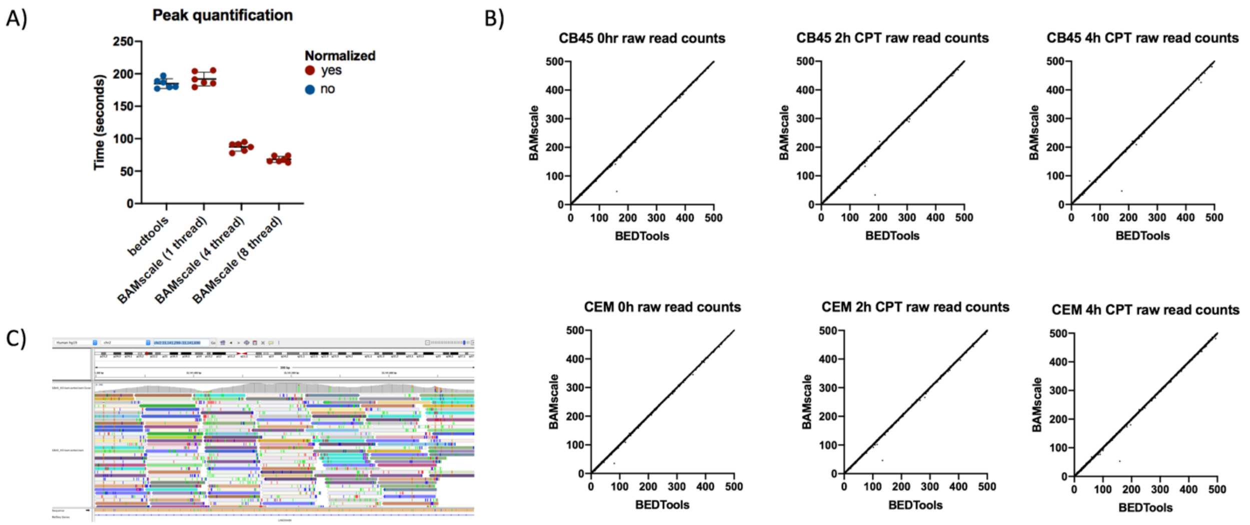

Supplementary figure 1. Benchmarking and comparison of different tools using ATAC-seq data. A) Peak quantification performance using *bedtools* and *BAMscale* (1, 4 and 8 execution threads). B) Comparison of raw read counts between *bedtools* and *BAMscale* in six ATAC-seq samples. C) IGV screenshot of a peak overestimated by *bedtools* in all samples, where read pairs align to different chromosomes.

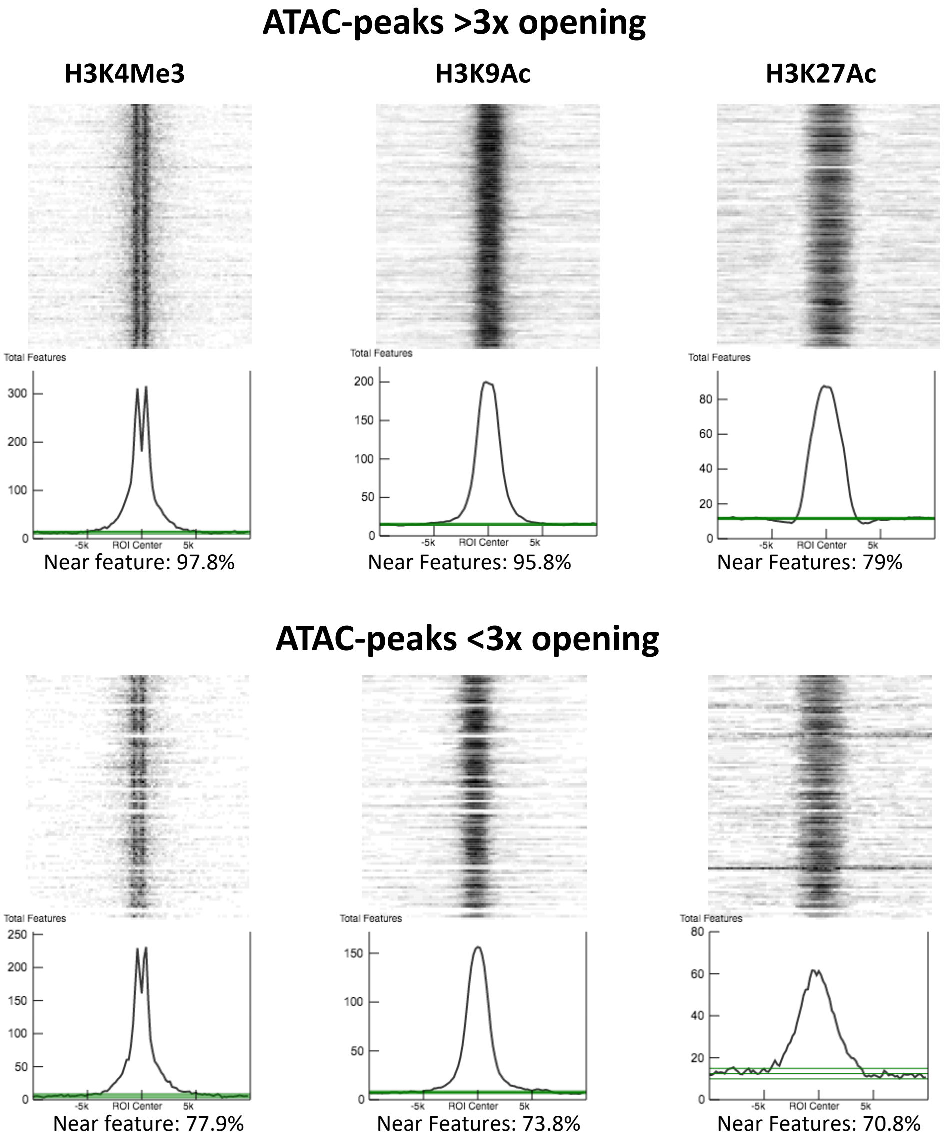

Supplementary figure 2. Colocalization of ATAC-seq peaks. Peaks with >3-fold opening had increased colocalization with H3K4me3 and H3K9ac compared to peaks with weaker or no increase.

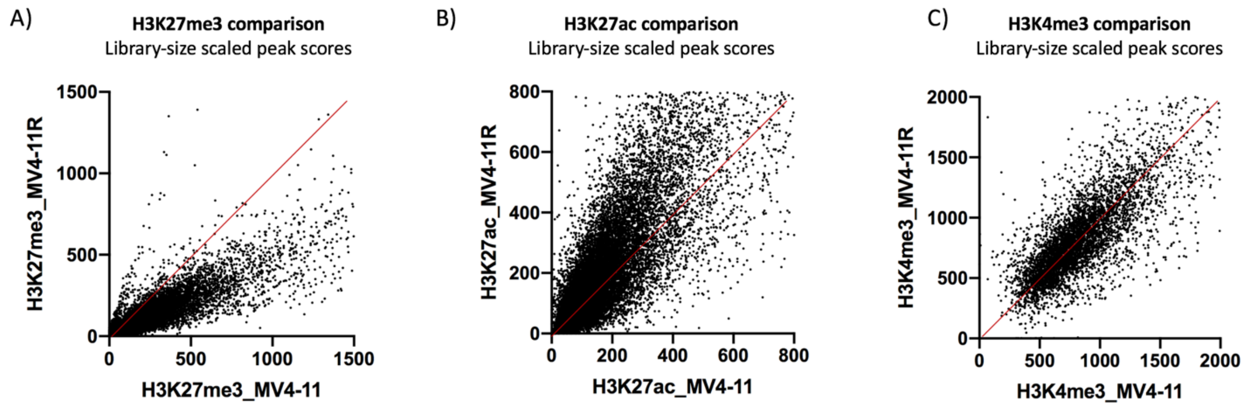

Supplementary figure 3. Changes in histone ChIP-seq signal between MV4-11 and MV4-11R cells. A) H3K27me3 signal decreased, B) H3K27ac signal increased, and C) H3K4me3 signal did not change in the MV4-11R cells compared to the MV4-11 cells.

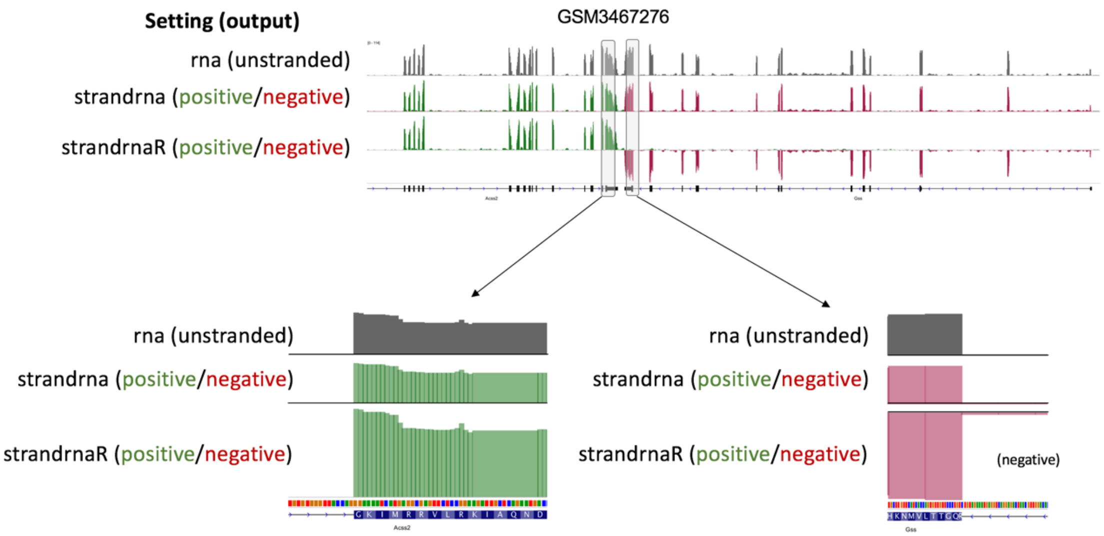

Supplementary figure 4. Stranded and unstranded RNA-seq coverage tracks created with BAMscale.

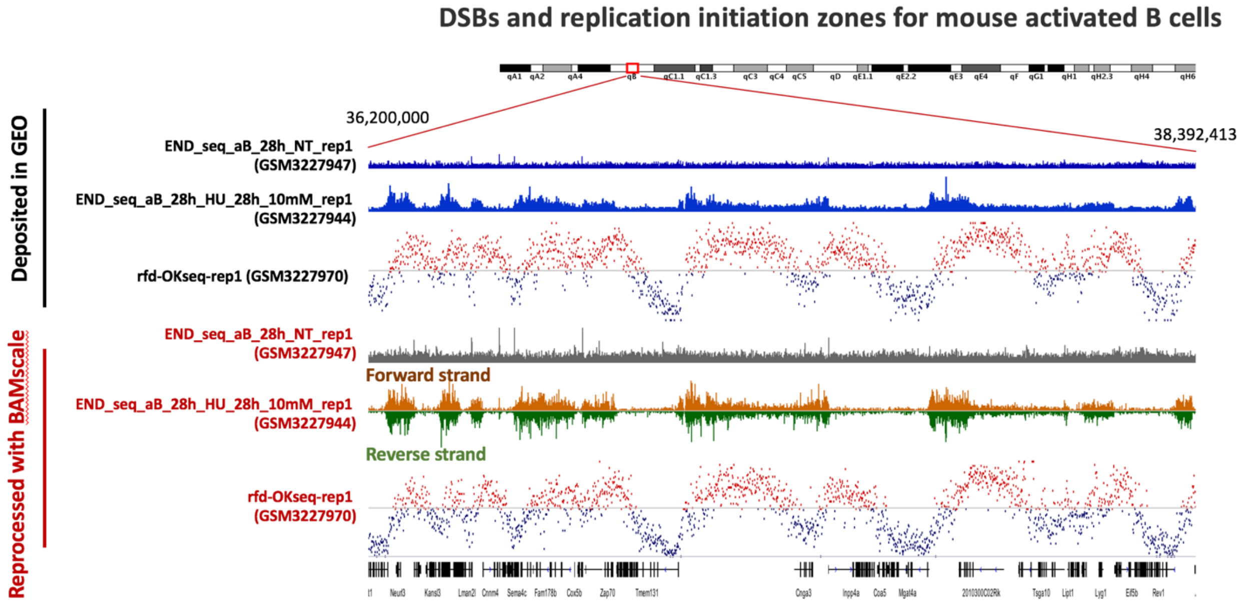

Supplementary figure 5. Comparison of deposited END-seq and OK-seq data reprocessed with BAMscale.

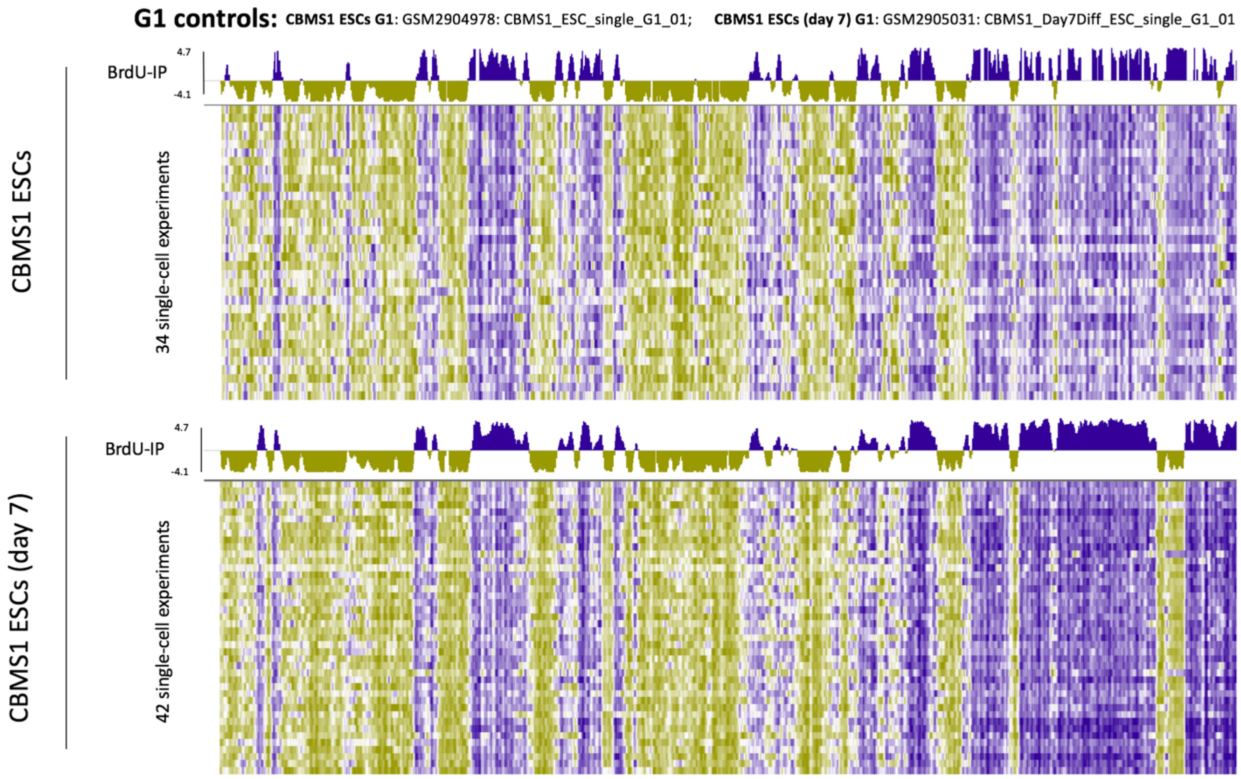

Supplementary figure 6. Reproducing replication timing from single-cell Repli-seq data.
